# Supplementary material for: A mixed methods evaluation of medication reconciliation in the primary care setting
Source: PLoS One. 2021 Dec 2;16(12):e0260882. doi: 10.1371/journal.pone.0260882 (PMC8638945; doi:10.1371/journal.pone.0260882)
Supplement: S1 File — (DOCX) [file pone.0260882.s001.docx]

*Appendix A*

**Clinic Observation Guide**

- Verify patient’s identity
- Clarification of allergies
- Access patient’s medical list
- Clarification of Pharmacy
- Review patient’s medication list with the patient
  - Use of go reconcile button

Notes:

- - Verify Patient’s current medication

Notes about patient knowledge:

- - - Are you using your medication as prescribed?
      - SIG (Dose, Route, Frequency, etc.) Change SIG, if applicable

Notes:

- - - Ask about new medication
      - Add new medications, if applicable
    - Ask about discontinued medications
      - Remove old medications, if applicable
    - Ask about held medication
      - Mark as not taking, if applicable
      - Reason discussed, if applicable
    - Delete duplicates, if applicable
  - Ask about side effects (adverse medication reactions)
    - Documentation of reaction
  - Ask about adherence (forgetfulness, trouble taking, or cost)
  - Ask about other prescription medications not listed
  - Ask about over the counter

Notes:

- - Mention of “as needed’’ medications
  - Ask about other concerns/questions
  - Make note to Physician, if applicable
  - Check mark as review
  - Ask if it was a normal day

*Appendix B*

**Interview Guide - Nurses**

**General Understanding of Demographics**

1. What is your role at Geisinger?
   1. Years in this role
      1. Previous experience

**Current Medication Reconciliation Practices**

1. Please walk me through your current rooming process focusing on how you discuss with patients their medication list.
2. Do you gather a patient’s medication list during every patient encounter?
3. When during the rooming process do you begin to collect the patient’s medication list?
4. What specific questions do you ask the patient?
   - 1. Do you tailor your approach for certain patients?
     2. Do you ask the same questions during every encounter?
     3. Prescription vs OTC?
     4. SIG?
        1. Name, dose, route, frequency, last taken, indication, and dosage form
     5. Inhalers/creams/ointments?
     6. Herbals/supplements/vitamins?
5. Please describe your interactions with the patient as part of this process.
   - 1. Challenges?
     2. Facilitators?
6. Please describe how you utilize Epic to record the patient’s medication list?
   - 1. What does Epic do well? Not so well?
     2. Do you know about or use the *Go Reconcile* button for medication lists?
7. What additional resources do you use to help you collect a patient’s medication list?
   - 1. Patient’s medicine vials, patient written medicine list, family members of patients, other medical records, etc.
8. How do you communicate with the provider about the patient’s medication history or any discussed patient issues?
   - 1. Do you inform the provider of added or discontinued meds, side effects, adherence, etc.
9. Do you think it is part of your role as a nurse to ask about medication side effects or adherence with patients?

**Knowledge of Medication Reconciliation**

I want to know your general thoughts about medication reconciliation.

1. What does medication reconciliation mean to you?
   1. Medication History?
2. What is your specific role in the medication reconciliation process?
   1. For your role, which term (Med Rec or Med history) do you prefer?
3. What training have you received on medication reconciliation/history? (rec or history based on answer to above question)

**General Feelings of Medication Reconciliation**

I want to know your general feelings about medication reconciliation.

1. What do you think are the benefits of medication reconciliation?
2. What are some challenges you face with collecting an accurate medication history?
   1. Technological barriers *(i.e. Epic)*?
   2. Workflow challenges *(i.e. time)?*
   3. Patient challenges?
   4. Other?

**Improvements to the Current Process**

I am trying to understand how to improve medication reconciliation/history.

1. What would a perfect medication reconciliation/history with a patient look like to you?

*(Despite previously mentioned barriers)*

- 1. What are indicators of a well-done medication history/reconciliation?
     1. How can we measure these indicators?

1. What improvements can be made to the current process?
2. In an observational sampling of 39 nurses and 166 patient care visits conducted this past summer by our staff in our primary care clinics during medication history taking we noted that only 37% of nurses ask a patient about over the counter medication use.
   1. What is your reaction?
   2. What percent of patients should be asked about OTC medication use in primary care?
   3. How would you suggest this could be improved?

**Other**

1. Do you have any other thoughts you would like to share?

Thank you for participating today. We are trying to get a comprehensive understanding of medication history taking and reconciliation at Geisinger. Is there anyone else that we should contact to who might be able to provide additional insight into this process?

**Interview Guide - Providers**

**General Understanding of Demographics**

1. What is your role at Geisinger?
   1. Years in this role
      1. Previous experience

**Knowledge of Medication Reconciliation**

I want to know your general thoughts about medication reconciliation.

1. What does medication reconciliation mean to you?
   1. Medication History?
2. What is your specific role in the medication reconciliation process?
   1. For your role, which term (Med Rec or Med history) do you prefer?
3. What training have you received on medication reconciliation/history? (rec or history based on answer to above question)

**Current Medication Reconciliation Practices**

1. Please walk me through how you talk with patients about their medication list.
2. Do you thoroughly review the patient’s medications during every encounter?
   - 1. What types of patients warrant a more thorough medication review? How do you make this decision?
3. When during the visit do you review patient’s medications?
4. How do your nurses communicate about patient’s medications with you?
   - 1. The nursing note, medication list comments, verbal comments, instant message, none, etc.
5. Please describe your interactions with the patient as part of this process.
   - 1. Challenges?
     2. Facilitators?
     3. Have you ever identified a discrepancy on a patient’s medication list? Can you please describe the situation?
6. Please describe how you utilize Epic to reconcile a patient’s medication list?
   - 1. What does Epic do well? Not so well?
7. What other resources do you use to ensure your patient has an accurate medication list?

**General Feelings of Medication Reconciliation**

I want to know your feelings about medication reconciliation.

1. What are the benefits of medication reconciliation?
2. What are some challenges with the current process?
   1. Technological barriers *(i.e. Epic)*?
   2. Workflow challenges *(i.e. time)?*
   3. Patient challenges?
   4. Other?

**Improvements to the Current Process**

I am trying to understand how to improve medication reconciliation.

1. What would a perfect medication reconciliation with a patient look like to you?

*(Despite previously mentioned barriers)*

1. What are indicators of a well-done medication history/reconciliation?
   - 1. How can we measure these indicators?
2. What improvements can be made to the current process?

**Other**

1. Do you have any other thoughts you would like to share?

Thank you for participating today. We are trying to get a comprehensive understanding of medication history taking and reconciliation at Geisinger. Is there anyone else that we should contact to who might be able to provide additional insight into this process?

**Interview Guide – Key Stakeholders (Informaticians, Managers, Pharmacists, etc.)**

**General Understanding of Demographics**

1. What is your role at Geisinger?
   1. Years in this role
   2. Responsibilities
2. Do you currently see patients?

**Knowledge of Medication Reconciliation**

I want to know your general thoughts about medication reconciliation.

1. What does medication reconciliation mean to you?
   1. Medication history?
2. What is your specific role in the medication reconciliation process?
   1. For your role, which term (Med Rec or Med history) do you prefer?
3. What training have you received on medication reconciliation/history? (rec or history based on answer to above question)

**Current Medication Reconciliation Practices**

1. Provider Process (to ask provider stakeholders)
2. Please walk me through how you talk with patients about their medication list.
   - 1. Do you thoroughly review the patient’s medications during every encounter?
        1. What types of patients warrant a more thorough medication review? How do you make this decision?
     2. When during the visit do you review patient’s medications?
     3. How do your nurses communicate about patient’s medications with you?
        1. The nursing note, medication list comments, verbal comments, instant message, none, etc.
     4. Please describe your interactions with the patient as part of this process.
        1. Challenges?
        2. Facilitators?
        3. Have you ever identified a discrepancy on a patient’s medication list? Can you please describe the situation?
     5. Please describe how you utilize Epic to reconcile a patient’s medication list?
        1. What does Epic do well? Not so well?
     6. What other resources do you use to ensure your patient has an accurate medication list?
3. Nurse Process (to ask nurse stakeholders)
   1. Please walk me through your current rooming process focusing on how you discuss with patients their medication list.
      1. Do you gather a patient’s medication list during every patient encounter?
      2. When during the rooming process do you begin to collect the patient’s medication list?
      3. What specific questions do you ask the patient?
         1. Do you tailor your approach for certain patients?
         2. Do you ask the same questions during every encounter?
         3. Prescription vs OTC?
         4. SIG?
            1. Name, dose, route, frequency, last taken, indication, and dosage form
         5. Inhalers/creams/ointments?
         6. Herbals/supplements/vitamins?
      4. Please describe your interactions with the patient as part of this process.
         1. Challenges?
         2. Facilitators?
      5. Please describe how you utilize Epic to record the patient’s medication list?
         1. What does Epic do well? Not so well?
         2. Do you know about or use the *Go Reconcile* button for medication lists?
      6. What additional resources do you use to help you collect a patient’s medication list?
         1. Patient’s medicine vials, patient written medicine list, family members of patients, other medical records, etc.
      7. How do you communicate with the provider about the patient’s medication history or any discussed patient issues?
         1. Do you inform the provider of added or discontinued meds, side effects, adherence, etc.
      8. Do you think it is part of your role as a nurse to ask about medication side effects or adherence with patients?

**General Feelings of Medication Reconciliation**

I want to know your thoughts about medication reconciliation.

1. What are the benefits of medication reconciliation?
2. What are some challenges with the current process?
   1. Technological barriers *(i.e. Epic)*?
   2. Workflow challenges *(i.e. time)?*
   3. Patient challenges?
   4. Other?

**Improvements to the Current Process**

I am trying to understand how to improve medication reconciliation.

1. What would a perfect medication reconciliation with a patient look like to you?

*(Despite barriers mentioned in previous process)*

1. What are indicators of a well-done medication history/reconciliation?
   - 1. How can we measure these indicators?
2. What improvements can be made to the current process?
3. In an observational sampling of 39 nurses and 166 patient care visits conducted this past summer by our staff in our primary care clinics during medication history taking we noted that only 37% of nurses ask a patient about over the counter medication use.
   1. What is your reaction?
   2. What percent of patients should be asked about OTC medication use in primary care?
   3. How would you suggest this could be improved?

**Other**

1. Do you have any other thoughts you would like to share?

Thank you for participating today. We are trying to get a comprehensive understanding of medication history taking and reconciliation at Geisinger. Is there anyone else that we should contact to who might be able to provide additional insight into this process?

*Appendix C*

**Interview Guide – Patients**

1. I have your survey answers in front of me, and I see in the survey you stated that it is *(survey answer #5)* that you are knowledgeable about what products you take to improve or maintain your health. Why do you feel you should be *(knowledgeable/not knowledgeable)*?
   - You also stated that it is *(survey answer #11)* that your healthcare provider knows about EVERYTHING you take to improve or maintain your health. Why do you feel your provider *(should/should not)* know about the medical products you take?
     - Probe on question 14 based on their response to question 11. This will vary per patient.
2. Again, according to the survey, you stated you *(do/do not (#9))* currently have an up-to-date list of what products you take to improve or maintain your health. Is this correct?
   - *If they do*, where did you get this list?
     - What format is it in?
     - Do you update it frequently?
     - Do you carry it with you all the time or just to the doctors?
       1. Why do you carry it with you?
   - *If they do not,* why do you not carry a list?
     - Would you start to carry one if your doctor told you to?
     - Do you think it would be beneficial for you to carry one?
       1. Why or why not?
3. So, you just explained to me that you *(do/do not)* currently have an up-to-date list of what products you take to improve or maintain your health, and your survey also states that you *(survey answer #16)* bring in your medication bottles with you to your provider visits. Why do you *(survey answer #16)* bring them?
   - *If they bring them,* do you find it beneficial to bring your medications with you? Why?
   - *If they do not bring them,* tell me about any concerns you would have bringing in your medication bottles.
     - If you were asked to bring in your medication bottles to future appointments, are you willing to do so?
       1. *If they do not bring them currently, but are willing to,* what would be the best way to remind you to bring your medication bottles to a visit with your provider?
          1. For example, a phone call, reminder letter, etc.
4. (*Check MyGeisinger data pulled before proceeding)*

*If they have MyGeisinger,* Geisinger has a patient portal called, MyGeisinger. How often do you use MyGeisinger?

- - How do you use the MyGeisinger portal?
    - For example, what do you do in the portal?
  - Do you ever adjust your medication list using MyGeisinger?
    - For example, adding or discontinuing a medication?
    - *If yes,* when you go to your visit, are the changes you made through MyGeisinger reflected on your medication list?

*If they do not have MyGeisinger,* skip this question.

1. Right now, I’m going to ask you to think back to visits with your Geisinger healthcare team.
   - How often do they review your medication list with you?
     - When they do review your list, how do they ask about your medications?
       - Do they review each medication with you or only some of your medications?
2. You stated in the survey that you are *(survey answer #10)* that your healthcare provider has a complete, accurate, and up-to-date list of what products you take to improve or maintain your health. What makes you *(survey answer #10)* that they have an accurate medication list?
   - During the medication list review with your Geisinger provider, has the list Geisinger has in their system ever not been correct? Tell me about that.
     - What recommendations do you have for how nurses and doctors can do a better job with collecting an accurate medication list?
3. Probe any other survey questions/answers specific to this patient.
   - *Maybe probe specifically about discrepancies between important that provider knows everything and not important provider knows about something specific.*
4. Do you have any other thoughts you’d like to share today regarding your experience of discussing medications with nurses and physicians at your visits?
   - What about any other thoughts you’d like to share about your perspective around your medications?

*Appendix D*

**Patient Survey**

1. Do you currently take any medications, vitamins, supplements, herbals, or other products to improve or maintain your health?

☐Yes

☐No

If no, the survey ends here.

1. How often do you need to have someone help you when you read instructions, pamphlets, or other written material from your healthcare provider or pharmacy?

☐Never

☐Rarely

☐ About half of the time

☐Most of the time

☐Always

1. Are you responsible for taking your own medications? (e.g. do you organize your pills and take them yourself without help?)

Yes

No

- 1. If no, who helps you take and manage your medications?

Family Member

Caregiver

Friend

Other Click or tap here to enter text.

1. On average, how often do you visit a healthcare provider?

☐More than once a week

☐Once a week

☐A few times a month

☐Once a month

☐Every few months

Once a year

Less than once a year

In the following questions (5-18), we are going to ask you about products that you take to improve or maintain your health, which could include prescribed medications, over-the-counter medications, vitamins, herbal or dietary supplements, creams, ointments, pastes, or other products applied to your skin, drops, sprays, or other products used on your eyes, ears, or nose.

1. How important is it to you that you are knowledgeable about what products you take to improve or maintain your health?

Not Important

Somewhat Important

Important

Very Important

1. I am confident I know what all of the products I take to improve or maintain my health are for.

☐Strongly Disagree

☐Disagree

☐Neither Disagree nor Agree

☐Agree

☐Strongly Agree

1. I can describe how to use or take all of the products that improve or maintain my health.

☐Strongly Disagree

☐Disagree

☐Neither Disagree nor Agree

☐Agree

☐Strongly Agree

1. How confident are you that you are able to tell a healthcare provider what products you take to improve or maintain your health?

☐Not Confident

☐Somewhat Confident

☐Confident

☐Very Confident

1. Do you currently have an up-to-date list of what products you take to improve or maintain your health?

Yes

No

1. How confident are you that your healthcare provider has a complete, accurate, and up-to-date list of what products you take to improve or maintain your health?

☐Not Confident

☐Somewhat Confident

☐Confident

☐Very Confident

1. How important is it to you that your healthcare provider knows about EVERYTHING you take to improve or maintain your health?

☐Not Important

☐Somewhat Important

☐Important

☐Very Important

1. Which of the following do you take to improve or maintain your health? (select all that apply)

Medications prescribed by my doctor or healthcare team

Medications available without a prescription (over-the-counter medication)

Vitamins

☐Herbal supplements

☐Dietary supplements

☐Other supplements

☐Creams, ointments, pastes, or other products applied to your skin

☐Drops, sprays, or other products used on your eyes, ears, or nose

☐Other Click or tap here to enter text.

1. How many different products, considering all categories listed above, do you take to improve or maintain your health: Insert dropdown box with options from 0-50+
2. How important is it that your healthcare provider knows about your use of:
   1. Medications prescribed by my doctor or healthcare team

☐Not Important

☐Somewhat Important

☐Important

☐Very Important

- 1. Medications available without a prescription (over-the-counter medication)

Not Important

Somewhat Important

Important

Very Important

- 1. Vitamins

Not Important

Somewhat Important

Important

Very Important

- 1. Herbal supplements

Not Important

Somewhat Important

Important

Very Important

- 1. Dietary supplements

Not Important

Somewhat Important

Important

Very Important

- 1. Other supplements

Not Important

Somewhat Important

Important

Very Important

- 1. Creams, ointments, pastes, or other products applied to your skin

Not Important

Somewhat Important

Important

Very Important

- 1. Drops, sprays, or other products used on your eyes, ears, or nose

Not Important

Somewhat Important

Important

Very Important

1. How often, when you visit your healthcare provider do you inform them about your use of the following:
2. Medications prescribed by my doctor or healthcare team

☐Never

☐Rarely

☐About half of the time

☐Most of the time

☐Always

1. Medications available without a prescription (over-the-counter medication)

Never

Rarely

About half of the time

Most of the time

Always

1. Vitamins

Never

Rarely

About half of the time

Most of the time

Always

1. Herbal supplements

Never

Rarely

About half of the time

Most of the time

Always

1. Dietary supplements

Never

Rarely

About half of the time

Most of the time

Always

1. Other supplements

Never

Rarely

About half of the time

Most of the time

Always

1. Creams, ointments, pastes, or other products applied to your skin

Never

Rarely

About half of the time

Most of the time

Always

1. Drops, sprays, or other products used on your eyes, ears, or nose

Never

Rarely

About half of the time

Most of the time

Always

1. How often do you bring the products you use to improve or maintain your health to visits with your healthcare provider?

Never

Rarely

About half of the time

Most of the time

Always

- 1. If never, why?

No one has ever told me I should bring them in

I do not want to

I do not think it is important

I forget or have a hard time remembering to bring them in

I am afraid of losing them

☐I am afraid of having them taken away or stolen

Other Click or tap here to enter text.

1. How comfortable would you be bringing the products you use to improve or maintain your health to visits with your healthcare provider in the future?

Not Comfortable

Somewhat Comfortable

Comfortable

Very Comfortable

1. In the past 3 months, has lack of affordability stopped you from using any of the products you use to improve or maintain your health or had a negative impact on your ability to afford other things?

Never

Rarely

About half of the time

Most of the time

Always

1. Would you be willing to be contacted to discuss your answers further with a researcher at Geisinger?

☐ Yes

☐ No

1. If yes, how would you like to be contacted?

☐ Email Click or tap here to enter text.

☐ Phone Click or tap here to enter text.

☐ Mail Click or tap here to enter text.

☐ Other Click or tap here to enter text.

*Appendix E*

**Staff Survey**

The following questions will mention the term medication reconciliation. Medication reconciliation is the process of ensuring that a patient’s medication list is complete and accurate. This process includes: 1. Collecting information from the patient and other sources, 2. Comparing that information to information held by the health system (typically in the electronic health record), and 3. Reconciling any differences.

Do you regularly conduct any component of medication reconciliation?

Yes

No

If no, survey ends here.

Do you work in the outpatient setting?

Yes

No

If no, survey ends here.

**Demographics** – This information will help us describe the participants that responded to the survey.

1. What is your age? (dropdown - Adjust lowest age to be 18)
2. What is your gender?

Male

Female

Other

Decline to Answer

1. Practice Area

Primary Care

Specialty Care

Other: Click or tap here to enter text.

- 1. If Specialty Care, which type?

65 Forward

Addiction Treatment

Allergies, Asthma, and Immunology

Anesthesiology

Breast Health

Cancer

Cosmetic Surgery

Dermatology

Diabetes

Ear, Nose and Throat (ENT)

Emergency Care

Endocrinology

Gastroenterology (GI)

Heart Care

Hepatology (Liver)

Home Health

Hospice

Joint Pain

Liver Care Center

Nephrology (Kidney)

Neurology & Neurosurgery

Optometry & Ophthalmology

Orthopedics & Sports Medicine

Pain Medicine

Palliative & Supportive Care

Pediatrics

Pharmacy

Physical Medicine and Rehabilitation

Plastic Surgery

Pregnancy and Newborn Care

Podiatry

Psychiatry & Behavioral Health

Pulmonology

Radiology

Rheumatology

Senior Health

Sleep Services

Surgery

Transplant Services

Urgent Care (Convenient Care)

Urology

Vascular Disease

Weight Loss

Women's Health

Other: Click or tap here to enter text.

1. Position

Case Manager

Community Health Assistant

Licensed Practical Nurse

Medical Assistant

Nurse Practitioner

Pharmacist

Physician Assistant

Physician Specialist

Primary Care Physician

Registered Nurse

Other: Click or tap here to enter text.

1. Years in position? (dropdown)

**Attitudes towards Medication Reconciliation**

1. How important is medication reconciliation in the patient care process?

Not important

Somewhat Important

Important

Very Important

1. How important is it that medication reconciliation occurs at every visit?

Not important

Somewhat Important

Important

Very Important

For the next three questions, please rate your agreement with the following statements.

1. The process of medication reconciliation is standardized across Geisinger.

Strongly Disagree

Disagree

Neither Agree nor Disagree

Agree

Strongly Agree

1. Having a standardized process for medication reconciliation across Geisinger would be beneficial.

Strongly Disagree

Disagree

Neither Agree nor Disagree

Agree

Strongly Agree

1. I have a well-defined role and know what I am responsible for in the medication reconciliation process.

Strongly Disagree

Disagree

Neither Agree nor Disagree

Agree

Strongly Agree

1. Which of the following parts of medication reconciliation do you do on a regular basis? (select all that apply)

Collect information from the patient and other sources

Compare to an existing list (e.g. in the electronic health record)

Reconcile any differences

Other: Click or tap here to enter text.

**Comfort with Medication Reconciliation**

1. How comfortable are you with your role in medication reconciliation?

Not Comfortable

Somewhat Comfortable

Comfortable

Very Comfortable

1. How comfortable are you adding medications to a patient’s medication list while conducting medication reconciliation?

Not Comfortable

Somewhat Comfortable

Comfortable

Very Comfortable

1. How comfortable are you removing medications from a patient’s medication list while conducting medication reconciliation?

Not Comfortable

Somewhat Comfortable

Comfortable

Very Comfortable

1. For the next question, please rate your agreement with the following statements.
   1. I do not believe it is my responsibility to conduct medication reconciliation.

Strongly Disagree

Disagree

Neither Agree nor Disagree

Agree

Strongly Agree

- 1. There are unclear guidelines for what I can and cannot remove from patients’ medication lists during medication reconciliation.

Strongly Disagree

Disagree

Neither Agree nor Disagree

Agree

Strongly Agree

- 1. I am uncomfortable conducting medication reconciliation due to my limited knowledge of medications.

☐Strongly Disagree

☐Disagree

☐Neither Agree nor Disagree

☐Agree

☐Strongly Agree

- 1. I am uncomfortable removing medications I did not prescribe/are not in my area of expertise.

Strongly Disagree

Disagree

Neither Agree nor Disagree

Agree

Strongly Agree

**Experience with Medication Reconciliation**

1. While conducting medication reconciliation have you ever found an error which had the potential to cause harm to the patient?

Yes

No

1. While conducting medication reconciliation have you ever identified an error which you believe did cause harm to the patient?

Yes

No

1. How often do you identify errors on patients’ medication lists?

Never

Rarely

About half the time

Most of the time

Always

1. After you finish conducting medication reconciliation, how confident are you that a patient’s medication list is an accurate reflection of the medications they are taking?

Not Confident

Somewhat Confident

Confident

Very Confident

1. How often do patients bring in their medication bottles from home to visits?

Never

Rarely

About half the time

Most of the time

Always

1. Do you ask patients to bring in their medication bottles from home to visits?

☐Yes

☐No

1. How often do patients bring in a medication list from home to visits?

Never

Rarely

About half the time

Most of the time

Always

1. If patients were to bring their medication bottles or a medication list to visits, it would help me in my role of conducting medication reconciliation.

Strongly Disagree

Disagree

Neither Agree nor Disagree

Agree

Strongly Agree

**Barriers to Medication Reconciliation**

1. How often do you encounter the following barriers to conducting medication reconciliation:
   1. Patients are not knowledgeable about their medications

☐Never

☐Rarely

☐ About half of the time

☐Most of the time

☐Always

- 1. I do not have time to conduct a thorough medication reconciliation/other tasks take priority

☐Never

☐Rarely

☐ About half of the time

☐Most of the time

☐Always

- 1. Patients do not want to participate

☐Never

☐Rarely

☐ About half of the time

☐Most of the time

☐Always

- 1. There are language barriers between myself and some patients

☐Never

☐Rarely

☐ About half of the time

☐Most of the time

☐Always

- 1. Patients receive healthcare outside of Geisinger

☐Never

☐Rarely

☐ About half of the time

☐Most of the time

☐Always

- 1. Entering patient reported medication in the Electronic Health Record is difficult

☐Never

☐Rarely

☐ About half of the time

☐Most of the time

☐Always

- 1. Other: Click or tap here to enter text.

☐Never

☐Rarely

☐ About half of the time

☐Most of the time

☐Always

**Training on Medication Reconciliation**

1. Have you ever had a formal training focusing on medication reconciliation from Geisinger?

☐Yes

☐No

1. I would benefit from additional training on medication reconciliation.

Strongly Disagree

Disagree

Neither Agree nor Disagree

Agree

Strongly Agree

1. If medication reconciliation training were offered, which format would you most prefer?

Live/in-person (if possible)

GOALS course

FastFacts/Written Document

Skype/Video

Other: Click or tap here to enter text.

**Final Thoughts on Medication Reconciliation**

1. What other thoughts would you like to share about the medication reconciliation process? This can include challenges or suggestions for improvement. (optional) Click or tap here to enter text.

*Appendix F*

**Staff Interviewee Demographics**

| Interviewee | Sex | Role |
| --- | --- | --- |
| 1 | Male | Physician/Clinic Leadership |
| 2 | Female | Physician/ Clinic Leadership |
| 3 | Male | Physician/Clinic Leadership |
| 4 | Female | Nurse |
| 5 | Female | Case Manager |
| 6 | Male | Physician |
| 7 | Female | Nurse |
| 8 | Male | Physician |
| 9 | Male | Physician |
| 10 | Female | Nurse |
| 11 | Female | Physician/Clinic Leadership |
| 12 | Female | Nurse |
| 13 | Female | Nurse |
| 14 | Female | Nurse |
| 15 | Female | Clinic Leadership |
| 16 | Male | Physician/Informatician |
| 17 | Female | CRNP |
| 18 | Female | CRNP |
| 19 | Female | Physician/Clinic Leadership |
| 20 | Female | MedRec Coordinator/Pharmacist |
| 21 (joint interview) | Female | Operations |
| 21 (joint interview) | Female | CMA/Operations |
| 22 | Female | Nurse |
| 23 | Female | Nurse |
| 24 | Male | Pharmacist/Informatician |
| 25 | Male | Physician |
| 26 | Female | Pharmacist/Pharmacy Leadership |
| 27 | Female | Pharmacist/Pharmacy Leadership |
| 28 | Male | Pharmacist/Pharmacy Leadership |
| 29 | Male | Physician Assistant |
| 30 | Male | Physician |
| 31 | Male | Pharmacist/Pharmacy Leadership |
| 32 | Female | Physician |
| 33 | Male | Pharmacist/Pharmacy Leadership |
| 34 | Male | Physician |
| 35 | Female | Pharmacist/Pharmacy Leadership |
| 36 | Male | Physician Specialist |
| 37 | Female | Nurse/Operations |
| 38 | Female | Informatician/Operations |
| 39 | Male | Physician |
| 40 | Male | Physician Assistant |
| 41 | Female | Case Manager/Leadership |
| 42 | Female | Pharmacist |
| 43 | Female | Pharmacist |
| 44 | Female | Nurse |
| 45 | Female | Physician |
| 46 | Male | Physician |
| 47 | Male | Physician Specialist |
| 48 | Female | Pharmacist/Pharmacy Leadership |

*Appendix G*

**Patient Interviewee Demographics**

| Interviewee | Age | Race | Sex | Number of Medications | Charlson Comorbidity Index Score |
| --- | --- | --- | --- | --- | --- |
| 1 | 70 | White | Female | 8 | 3 |
| 2 | 76 | White | Male | 23 | 7 |
| 3 | 54 | White | Male | 18 | 2 |
| 4 | 79 | White | Female | 23 | 12 |
| 5 | 61 | White | Male | 2 | 2 |
| 6 | 71 | White | Male | 1 | 4 |
| 7 | 60 | White | Female | 15 | 3 |
| 8 | 69 | White | Male | 15 | 2 |
| 9 | 65 | White | Male | 2 | 2 |
| 10 | 59 | White | Female | 5 | 2 |

*Appendix H*

**Staff Survey Flow Chart**

Survey sent out to eligible staff

N=2,541

Eligible staff survey responses

N=616

(24% completion rate)

Incomplete surveys of only demographic data

N=17

Consented to participate

N=633

No response to the survey

N=1889

Did not consent to participate

N=19

*Appendix I*

**Patient Survey Flow Chart**

Survey sent out to eligible patients

N=5,132

No response to the survey

N=4,429

Did not consent to participate

N=79

Consented to participate

N=624

Incomplete surveys

N=47

Eligible patient survey responses

N=577

(11% completion rate)
