# Supplementary material for: A mixed methods evaluation of medication reconciliation in the primary care setting
Source: PLoS One. 2021 Dec 2;16(12):e0260882. doi: 10.1371/journal.pone.0260882 (PMC8638945; doi:10.1371/journal.pone.0260882)
Supplement: Attachment — Submitted filename: Response to reviewers.docx [file pone.0260882.s002.docx]

**Response to Reviewers**

On behalf of the authors, I would like to thank the editor and reviewers for their suggestions. We have attempted to address these suggestions through a significant re-write and re-organization of the manuscript. Please find below a point-by-point response to the concerns that were raised.

Sincerely,

Michael Gionfriddo

**Comments from the Academic Editor**

*I concur with the general tenor of the reviewer's comments. In what I think is an attempt to be rigorous, the authors have employed a number of research methods that do not clearly link together. This, in turn, returns a significant amount of data - and the authors appear to report back on so many data points that the paper becomes very difficult to read. The authors should consider deciding what is nice versus what is needed in both their methods and results.*

Response

After careful consideration we have completely re-organized the results section and in the process significantly shortened the manuscript. We hope this has added the clarity and conciseness sought by the editor. We however, are concerned about shortening the methods section as we believe they are important for readers to understand any potential biases or limitations in our approach.

**Comments from Reviewer 1**

Comment 1

*Thank you for the opportunity to review this manuscript. Upon a full reading, I do not believe that this paper does justice to the excellent opportunity this study had to collect comprehensive mixed-methods data on the topic of medication reconciliation in the outpatient setting; nor does it do justice to analyzing and presenting the mixed-method data collected.*

*I see several opportunities for major revision that could serve to strengthen the readability and impact of this manuscript.*

*• A major concern is that this paper does not provide the BIG PICTURE on study findings to address its RESEARCH QUESTIONS (and the latter in turn are not clearly stated in the INTRODUCTION). It appears that the main objective is to understand barriers to effective medication reconciliation in the outpatient setting, with special attention to barriers to obtaining an accurate medication history upon patient arrival to the clinic.*

*• There is no alignment across manuscript sections to address the study aims/research questions.*

Response

We have updated the objective based on reviewers’ feedback. The objective now reads: To understand the extent to which behaviors consistent with high quality medication reconciliation occurred in primary care settings and explore barriers to high quality medication reconciliation. We have also added the specific research questions the study aimed to answer which were:

1. To what extent are behaviors indicative of a best possible medication history for medication reconciliation adhered to in a primary care setting?

2. What are the barriers which inhibit the accurate completion of medication reconciliation? Do these barriers differ between patients and staff or between staff with and without prescribing authority?

3. What changes do patients and staff think can be made to improve the process of medication reconciliation in primary care?

At the beginning of the discussion we tie our findings back to these research questions to hopefully improve upon the lack of alignment noted by the reviewer.

Comment 2

*• A logical beginning to addressing such a research question, would be to describe the EXISTING LITERATURE on influencing factors or barriers/facilitators to effective medication reconciliation and identify GAPS that the paper would be addressing. For example, there may be a gap in understanding the med history process from the perspective of both providers and patients.*

Response

We have modified the introduction to better highlight the gaps in the existing literature.

Comment 3

*• Next, the METHODS that will be used to address the research questions should be clearly described. Presently, all three methods appear to be disparate in addressing different objectives. Data collected on adherence to best practices during observation does not appear to feed into interview or survey data collection in any meaningful way that also ties back to both the existing literature and research questions. Likewise, findings from survey are not integrated into findings from interviews in any meaningful way.*

Response

We decided to present the methods separately for clarity, however we clearly indicate how each method contributed to the development of the study design. In the results however, the findings from the interviews, surveys, and observations are triangulated to present a more complete picture of the process of and barriers to medication reconciliation. Overall, across each of the methods the findings were concurrent and reinforced each other. In response to the reviewer’s comment, we have tried to tie our findings to additional literature within the discussion.

Comment 4

*• If the authors feel that they did not have a representative sample of patients from survey, then survey data from patients should not be included. Likewise, the patient sample interviewed appears to be too small to make meaningful distinctions between patients with fewer than 5 meds, patients with 5-10 meds and patients with greater than 10 meds, which would be very important to know from perspective of accurate medication history. It also does not help to distinguish between patients by level of empowerment or health literacy which would be very relevant to the problem being examined. The section on ‘patient-related barriers,’ page 27 appears to have been developed almost entirely from staff input. If the investigators feel that the interviewed patient sample is not representative, this component should not be included in the study.*

Response

As part of responding to reviewers comments, we have completely reorganized the results section including the themes and subthemes. As a result, we have removed the section on patient related barriers and integrated those into the relevant themes where appropriate. In this way the patient data is used to help triangulate the themes which were identified by the staff. We concur that further research is needed on patient barriers and perceptions of medication reconciliation and have added a statement to the discussion saying so.

Comment 5

*• Presently, there is no explanation of how each of the three “sequential” methods complemented each other to provide a holistic understanding of the problem of interest to address the research question. I would recommend reviewing the book on mixed method research design by Creswell and Creswell (2018) for tips on presenting results from mixed-methods research.*

Response

We understand how it may not have been clear how our methods complemented each other. To clarify we have added an additional paragraph to the methods which reads: We conducted a fully mixed sequential equal status mixed methods[16] evaluation of medication reconciliation within ambulatory care clinics combining ethnographic observations, semi-structured interviews, and surveys. The observations occurred first and informed both survey and interview guide development. Subsequently, we performed staff interviews from which we identified themes that led us to create targeted questions to assess in the staff survey to understand the applicability of the themes to the broader population of staff at our institution. The patient survey, which was informed by the observations and interviews with staff was then used to recruit patients to interview. The findings from each method are mixed in the results based on thematic categorization and act as a form of triangulation.

Comment 6

• *There are also multiple issues with the presentation of RESULTS.*

*• The presentation of qualitative results begins abruptly on pages 10 and 11 with no preparation for the reader. There are no subheadings related to thematic categories on these pages and then we see the first category on ‘staff-related’ barriers introduced on page 12. Frankly, the thematic categories of staff and patient related barriers appear to be weak, and not impactful in providing insight into strategies for overcoming barriers identified to accurate medication history.*

Response

We have substantially modified the presentation of the results based on reviewer feedback. We have moved away from the framework of staff and patient barriers and there is now an overarching theme (Inconsistency) with introductory text to guide the reader in the presentation of the results.

Comment 7

• *Moreover, the categorization of ‘technological barriers’ under ‘logistical problems’ does not appear to do justice to the critical problem which the write-up appears to be depicting, i.e., a MISMATCH BETWEEN CLINICAL WORKFLOW AND EHR SYSTEM DESIGN, which in turn can provide insight into impactful strategies for process and system redesign for med history process on EHR.*

Response

We thank the reviewer for this comment and as part of a restructuring of themes have incorporated the reviewer’s suggestion. There is now a theme around inadequate integration of medication reconciliation into clinical workflows which includes issues with the electronic health record.

Comment 8

*• Following form the above concern, there needs to be more transparency related to the coding methodology used for qualitative (thematic) analysis. How were these categories identified, and how do they tie back to the existing literature on barriers already identified, and how can they be leveraged to gain insight into strategies for improvement?*

Response

We have clarified in the text that the coding was inductive and that themes were collaboratively generated based on discussion with the team upon reviewing the inductively identified codes. Additionally, we have added additional discussion about how some of the themes tie back to existing literature as well as better clarifying potential areas for improvement based on our findings.

Comment 9

*• Overall, the paper gets caught up in the DETAILS of the qualitative study with a preponderant focus on presenting detailed comments from staff and patient interviews (which is frankly fatiguing to the reader, since it is a mishmash of comments that could belong in any of the thematic categories specified). If the paper is going to focus primarily on qualitative data obtained from staff interviews, then I would recommend a full revision of the presentation of results to focus on the BIG PICTURE findings related to barriers to effective med history, with a supporting table on DETAILS from qualitative comments.*

Response

We have substantially revised and reorganized the manuscript to improve readability and focus including the removal or paring down of several quotes. We believe the remain quotes are more focused and help to ground the themes in the data which adds credibility to our findings.

Comment 10

*• In a similar vein, the DISCUSSION section references interventions that have been implemented in the literature, however, the discussion needs to provide a clear summary of findings, discuss limitations and then outline a clear set of implications from the study for practice and future research, including insights into strategies and interventions for overcoming barriers identified to med history identified in the study. Likewise, the CONCLUSION needs to be strengthened to more impactful in conveying the overall contribution of the study to the literature and to addressing the problem of interest.*

*In summary, there are many substantial insights that could emanate from this study, on both barriers to med history and strategies for overcoming them, but it will require a major revision of this manuscript to flesh these out and realize the full potential of the study.*

*Thank you for the opportunity to review this manuscript.*

Response

In response to the reviewers comment, we have substantially revised the discussion including a new summary of the results and conclusion.

**Comments from Reviewer 2**

Comment 1

*This research is a very useful contribution to an important area of clinical practice, thank you. Investigation of medication reconciliation practices and attitudes and behaviors in primary care settings is identified as a gap in the literature.*

*A mixed methods approach to describe and explore this topic is appropriate, and may be useful to generate hypotheses for future research.*

*My concerns with this manuscript are primarily focused on the way in which the study is described and presented.*

*1. The stated aim of the study is:*

*'To understand the extent to which behaviors consistent with high quality medication*

*reconciliation occurred in primary care settings and which factors contribute to*

*inaccurate medication lists', however that aim does not appear to be achieved with the study as described.*

*The work that has been presented does explore practices/behaviors and attitudes, but is not designed to '...understand the extent to which behaviors consistent with .... contribute to inaccurate medication lists.'*

*I suggest that the study objective be reworded to align with the study methods as presented in the paper.*

Response

Based on the reviewer’s feedback, we have revised our objective statement to more accurately reflect the aim of our study which now reads: “To understand the extent to which behaviors consistent with high quality medication reconciliation occurred in primary care settings and explore barriers to high quality medication reconciliation.”

Comment 2

*2. In the first sentence of the paper (Line 44) "Failure to conduct proper medication reconciliation results in....' - the use of the word 'proper' is unusual in a scientific paper. While this may be conversational and understood, it would be more appropriate to consider other terms. For example, does this mean- inaccurate, undocumented, incomplete, absent…?*

Response

We have changed this fragment to now read: “Inaccurate or incomplete medication reconciliation…”

Comment 3

*3. Definition of medication reconciliation. The reader would expect to see the precise definition of the term, as used by the authors in this research given the nature of the study. The literature has used this term widely. Furthermore the audience for this journal may not have a single interpretation. In the manuscript it is stated: (line 45) ‘Medication reconciliation is the process of creating an accurate medication list'. This might be considered a limited definition, given the work in the manuscript.*

*The authors have referenced a paper (#5) that does provide a comprehensive statement:*

*"... a proposed definition for medication reconciliation tasks as "the process of creating the most accurate list possible of all medications a patient is taking and comparing that list against the prescriber's orders. In addition, the patient's allergies, history of side effects from medications and medication aids are listed with the goal of providing correct medication to the patient at all transition points within the health care system."*

*It would seem key to include a definitive statement of medication reconciliation in the introduction to this paper.*

Response

We have now added the full definition of medication reconciliation to the manuscript.

Comment 4

*4. The sentence in lines 70-72 appear incomplete, a typographical error.*

*"The observations were considered quality improvement (IRB#2019-0561) while consent was obtained for the surveys and interviews and deemed exempt (IRB# 2019-0868)"*

Response

To improve clarity, we have now revised this sentence to read: The observations were considered quality improvement (IRB#2019-0561), while the surveys and interviews were deemed exempt (IRB# 2019-0868).

Comment 5

*5. Line 79: 'rooming staff' – this term may need definition for an international audience.*

Response

Thank you for this comment, we have replaced rooming staff with “nurse or medical assistant”

Comment 6

*6. Line 164: "All statistical analysis was done with SAS." Rather than identifying the software product, please outline the statistical tests/methods/analyses that were utilized to address the specific quantitative research aims*.

Response

We have added additional detail on the statistical analysis. The section now reads: Comparisons of continuous data were conducted using t-tests, while comparisons of categorical data were conducted using chi-square or Fisher’s exact tests as appropriate. All statistical analyses were done with SAS (SAS 9.4, Cary, NC) or R (The R Group, Vienna, Austria) and p-values <0.05 were considered significant; no adjustment was made for multiple comparisons.

Comment 7

*7. Line 665: "... a thorough mixed methods evaluation" The word ‘thorough’ is not needed, nor appropriate. If the authors intend to highlight the rigorous or comprehensive nature of their study, then that should be stated and justified. It appears that the intention here is to declare that this study is appropriately designed and conducted in such a way to meet the study objectives.*

Response

We have removed this adjective at the advice of the reviewer.
