# Supplementary material for: A mixed methods evaluation of medication reconciliation in the primary care setting
Source: PLoS One. 2021 Dec 2;16(12):e0260882. doi: 10.1371/journal.pone.0260882 (PMC8638945; doi:10.1371/journal.pone.0260882)
Supplement: Attachment — Submitted filename: Response to reviewers_revision.docx [file pone.0260882.s003.docx]

**Response to Reviewers**

On behalf of the authors, I would like to thank the editor and reviewers for their suggestions. We have attempted to address these suggestions of the reviewer. Please find below a point-by-point response to the concerns that were raised.

Sincerely,

Michael Gionfriddo

**Comments from Reviewer 1**

Comment 1

*This is a substantially improved version of the manuscript! I appreciate the authors’ high responsiveness to reviewer recommendations. I do however have some suggestions for minor revisions at this stage to improve the overall organization and flow of the manuscript, particularly in regard to strengthening the ALIGNMENT between research questions and presentation of methods, results, discussion and conclusion.*

We thank the reviewer for this comment. We have endeavored to improve the alignment based on their subsequent comments.

Comment 2

*METHODOLOGY*

*• It would be helpful to have a brief explanation of how the observation component of the methodology helped to develop the interview guide and likewise, how both observations and interviews contributed to development of the survey. Just a few key EXAMPLES would be helpful in illustrating the complementarity among the three methods in providing a holistic understanding of the problem (and addressing the three research questions).*

To clarify how the methods were mixed we have added a few examples in the text. (lines 84-93).

“The observations occurred first and informed both survey and interview guide development. For example, we noted in the observations that the adherence to best practices for conducting a BPMH were variable. To better understand this variability, we asked questions in the interview and surveys such as whether the staff had been trained in conducting medication reconciliation/BPMH. Similarly, in the observations we noted that over-the counter (OTC)/non-prescription medications were asked about infrequently so we specifically asked about this during the interviews. Following the observations, we performed staff interviews from which we identified themes that led us to create targeted questions to assess in the staff survey to understand the applicability of the themes to the broader population of staff at our institution. For example, during the interviews we noted that time emerged as a barrier and was thus included on the staff survey.”

Comment 3

• *It would also be helpful to clarify that Research Question 1 is addressed by observation, while Research Questions 2 and 3 are addressed through surveys and interviews*.

We have added language to clarify this in the methods section when discussing each method.

Comment 4

*RESULTS*

*• It would be helpful to have broad subsections corresponding to the three research questions in the Results to improve alignment. E.g., results related to: 1) medication reconciliation best practice behaviors; 2) barriers, and 3) suggestions for improvement.*

We appreciate this comment, but based on our analysis strategy (thematic analysis) we have chosen to present the data thematically (with themes derived from the data) with the barriers and suggestions discussed within each theme. In reviewing our notes, we had initially broken it out into each research question but during the process of synthesizing the data felt that a more thematic approach better tied the data together. Further, given the primacy of observations for addressing question 1, separating them out would reduce the extent to which our data were mixed and we believe that separation would detract from the manuscript. However, if the editor feels it is in the best interest of manuscript to reorganize, we will reconsider.

Comment 5

*• Line 287-288 in Track Changes version requires clarification. It says non-prescribers were more likely to agree there was a standardized process but the quotations do not corroborate this.*

We understand the reviewers’ confusion. While these seem contradictory it is not so. The majority of both prescribers and non-prescribers felt that there was a lack of a standardized process which aligns with the findings of our interviews (and illustrated in the quotes). Further review was unable to identify specific quotes which reflect an endorsement that a standardized process currently exists.

Comment 6

• *It would be most helpful to have a table summarizing the key results related to research question 2 (i.e., barriers) and research question 3 (i.e., corresponding suggestions for improvement). Even a simple listing of inconsistencies related to workflow, communication, documentation etc. (from perspective of staff/patient) with corresponding simple suggestions for improvement would greatly help to strengthen the presentation of results.*

*• The narrative would serve to supplement such a key results table in highlighting relevant quotations.*

Based on the reviewer’s comment we have created a new Table (Table 3) illustrating the main findings related to each research question and used this to replace the previous Table 1.

Comment 7

DISCUSSION

• *It would be important to elaborate on findings related to Research Question 3 (suggestions for improvement) in the Discussion (e.g., some details related to standardized workflows, education, training, EHR redesign etc.).*

We have added an additional paragraph describing suggestions in the discussion to address the reviewer’s concern.

“To address these barriers, staff and patients made several suggestions including improved education and training, reminders, workflow standardization and redesign, EHR redesign, and increased time or designated visits to conducted medication reconciliation. A major barrier we identified was lack of training (70% of staff reported not receiving training from the system on medication reconciliation). While discussions about medication reconciliation often occur in the didactic and experiential training of health professionals, training as part of onboarding not only emphasizes its importance and reinforces best practices, but could help to clarify responsibilities, increase comfort, and standardize the workflow across the system. Based on this potential, we are currently implementing and evaluating a training program for medication reconciliation within our system” (line 549-558)

Comment 8

CONCLUSION

• *It would be helpful to include a couple of sentences related to suggestions for improvement (research question 3) to provide a complete picture of the study contribution in the conclusion.*

*Again, I appreciate the authors’ efforts in revising this paper! Best wishes.*

We have added a few sentences to the conclusion to address the reviewers’ concern.

“Many possible solutions were noted by patients and staff. One example, education and training, can address several of these inconsistencies and is actively being evaluated within our system. We hope that by using challenges and potential solutions derived from our staff and patients that the findings will have increased salience and lead to sustained improvements in medication reconciliation.” (lines 632-636)
